# Structural and functional characterization of protein–lipid interactions of the *Salmonella typhimurium* melibiose transporter MelB

**DOI:** 10.1186/s12915-018-0553-0

**Published:** 2018-08-03

**Authors:** Parameswaran Hariharan, Elena Tikhonova, João Medeiros-Silva, Aike Jeucken, Mikhail V. Bogdanov, William Dowhan, Jos F. Brouwers, Markus Weingarth, Lan Guan

**Affiliations:** 1 Department of Cell Physiology and Molecular Biophysics, Center for Membrane Protein Research, School of Medicine, Texas Tech University Health Sciences Center, Lubbock, TX 79430 USA; 2 NMR Spectroscopy, Bijvoet Center for Biomolecular Research, Department of Chemistry, Faculty of Science, Utrecht University, Padualaan 8, 3584 CH Utrecht, The Netherlands; 3 Department of Biochemistry & Cell Biology, Lipidomics Facility, Faculty of Veterinary Medicine, Utrecht University, Yalelaan 2, 3584 CM Utrecht, The Netherlands; 4 Department of Biochemistry and Molecular Biology, the University of Texas Health Science, Center McGovern Medical School, Houston, TX 77030 USA

**Keywords:** Phospholipids, Sugar transport, Membrane protein, Substrate binding, Solid-state NMR, Mass spectrometry, Circular dichroism spectroscopy, Melting temperature

## Abstract

**Background:**

Membrane lipids play critical roles in the structure and function of membrane-embedded transporters. *Salmonella typhimurium* MelB (MelB_St_) is a symporter coupling melibiose translocation with a cation (Na^+^, Li^+^, or H^+^). We present an extensive study on the effects of specific phospholipids on the structure of MelB_St_ and the melibiose transport catalyzed by this protein.

**Results:**

Lipidomic analysis and thin-layer chromatography (TLC) experiments reveal that at least one phosphatidylethanolamine (PE) and one phosphatidylglycerol (PG) molecule associate with MelB_St_ at high affinities. Solid-state nuclear magnetic resonance (ssNMR) spectroscopy experiments confirmed the presence of lipid tails and glycerol backbones that co-purified with MelB_St_; headgroups of PG were also observed. Studies with lipid-engineered strains, including PE-deficient, cardiolipin (CL)- and PG-deficient, or CL-deficient strains, show that lack of PE or PG, however not CL, largely inhibits both H^+^- and Na^+^-coupled melibiose active transport to different extents. Interestingly, neither the co-substrate binding (melibiose or Na^+^) nor MelB_St_ folding and stability are affected by changing lipid compositions. Remarkably, the delipidated MelB_St_ with only 2–3 bound lipids, regardless of the headgroup species, also exhibits unchanged melting temperature values as shown by circular dichroism spectroscopy.

**Conclusions:**

(1) Lipid tails and glycerol backbones of interacting PE and PG may contribute to the stability of the structure of MelB_St_. (2) The headgroups of PE and PG, but not of CL, play important roles in melibiose transport; however, lipid headgroups do not modulate the folding and stability of MelB_St._

**Electronic supplementary material:**

The online version of this article (10.1186/s12915-018-0553-0) contains supplementary material, which is available to authorized users.

## Background

**Fig. 1:**
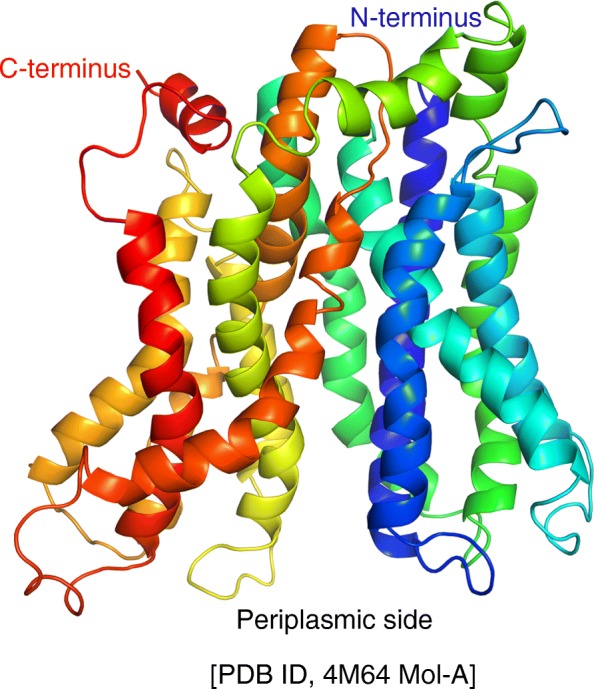
A 3-D crystal structure of MelB_St_ [pdb access ID, 4M64 Mol-A]. The overall fold of MelB_St_ in a periplasmic-side-open conformation. Helices are colored in rainbow colors from blue (N terminus) to red (C terminus). The cytoplasm-located N- and C-termini are labeled in blue and red text, respectively

Cell membranes form biological barriers that selectively allow specific ions and solutes to permeate. The functions of cell membranes rely on both lipids and proteins, as well as their interactions. *Salmonella typhimurium* MelB (MelB_St_) encoded by the *melAB* operon is a cation-coupled symporter with 12 transmembrane α-helices embedded in the cytoplasmic membrane [1–3]. This transporter catalyzes stoichiometric melibiose translocation across the membrane coupled to the transduction of the cations Na^+^, Li^+^, or H^+^ [1, 2, 4–6]. Melibiose active transport against concentration gradient is driven by an electrochemical H^+^, Na^+^, or Li^+^ gradient, while MelB can also catalyze melibiose downhill transport in the absence of these electrochemical ion gradients. MelB is a member of glycoside-pentoside-hexuronide:cation symporter (GPH) [7] belonging to the major facilitator superfamily (MFS) [8], a major group of transporters with similar overall fold that is ubiquitously found in all classes of organisms. The high-resolution X-ray 3-D crystal structure of MelB_St_ at a resolution of 3.35 Å [2] exhibits a protein fold typical of an MFS permease [9, 10] with two N- and C-terminal 6-helix bundles surrounding an aqueous cavity containing residues important for the binding of galactosides and Na^+^, Li^+^, or H^+^ [2]. Most transmembrane helices are heavily distorted with kinks (Fig. 1), and no lipid was resolved. An alternating-access mechanism was suggested to operate the transport by MelB [1, 2, 11–14], i.e., the permeases cycle different conformational states including at least an outward-open, inward-open, and occluded intermediate state. A recent study suggests that the cooperative binding of the cargo sugar and the coupling cation to MelB_St_ triggers the global conformational changes [15]. Since the protein is expected to undergo large conformational changes and span several conformational states, how these transport steps are affected by the surrounding lipids is unknown. It has been showed that an optimal melibiose active transport catalyzed by the MelB of *Escherichia coli* (MelB_Ec_) requires phospholipids containing a C16:1 acyl chain [16]. However, detailed structural and functional studies on the individual effects of lipid headgroups and tails on MelB are not available, and such information is also absent for most membrane transporters. The missing knowledge hinders our understanding of the membrane transport mechanism in general.

Bacterial membranes consist of various phospholipids. Structurally, each phospholipid contains two hydrophobic acyl chains (tail) and a hydrophilic headgroup, and the two components are linked by a glycerol backbone. Each headgroup contains a phosphorus and either an ethanolamine (i.e., phosphatidylethanolamine, PE), glycerol (i.e., phosphatidylglycerol (PG), or other groups. In *E. coli*, the cytoplasmic membrane consist of ∼ 69% PE, ∼ 19% PG, ∼ 6% cardiolipin (CL, a dimer of phosphatidic acids connected by a glycerol), and 5% few other lipid types [17]. This lipid composition is also found with *S. typhimurium* membranes [17]. The effects of lipid headgroups on membrane protein folding and activities have been extensively studied with the lactose permease of *E. coli* (LacY) [18–20]. PE was clearly shown to govern the membrane topology of LacY and to be essential for the H^+^-coupled uphill transport activity of LacY [18, 20, 21]. In the absence of PE, LacY molecules are inserted into the membrane at inverted orientation of transmembrane helices I-VI, and supplementation of PE in vivo [20, 22, 23], in situ [24], or in vitro [19], restores the overall protein folding to a nearly native topology, as well as lactose uphill transport activity. Notably, while PE is needed for LacY to carry out uphill accumulation of sugar substrates, it is not required for sugar gradient-driven downhill equilibration [18, 20, 25]. PE effects on membrane topology and function have been also described in other H^+^-coupled transporters including the phenylalanine permease PheP [26] and γ-aminobutyric acid permease GabP [27], and the members of amino acid-polyamine-organocation (APC) transporters. With the bacterial K^+^ channel KcsA, non-annular lipids were resolved between channel monomers in X-ray crystal structures [28], and solid-state NMR (ssNMR) spectroscopy studies [29] demonstrated that these tightly bound lipids feature anionic headgroups. Moreover, functional studies demonstrated that anionic lipids modulate KcsA activity [30, 31]. Recently, a structure of an osmotic stress-regulated betaine transporter BetP revealed five non-annular lipids at the BetP trimer center and three annular lipids at the trimer periphery [32], likely all PG, providing critical information on the roles of lipids in transport and regulation of BetP.

In this study, we applied an integrated approach, including ssNMR spectroscopy, mass spectrometry, circular dichroism (CD) spectroscopy, genetic engineering, and transport assays to analyze the effects of lipid headgroups and tails on MelB_St_ structure and function individually. We have identified at least one non-exchangeable PE and one non-exchangeable PG that tightly bind to MelB_St_. In the absence of PE or PG, the cation-coupled active transport against melibiose concentration is largely inhibited; however, no effect is obtained in the absence of CL. Furthermore, neither the folding and stability of MelB_St_ nor the binding affinity for its co-substrate melibiose and Na^+^ is affected by the identity on the lipid headgroups, which suggest that the lipid tails from only few tightly bound phospholipids can stabilize the MelB_St_ structure.

## Results

### Effect of zwitterionic PE on melibiose active transport by MelB_St_

**Fig. 2:**
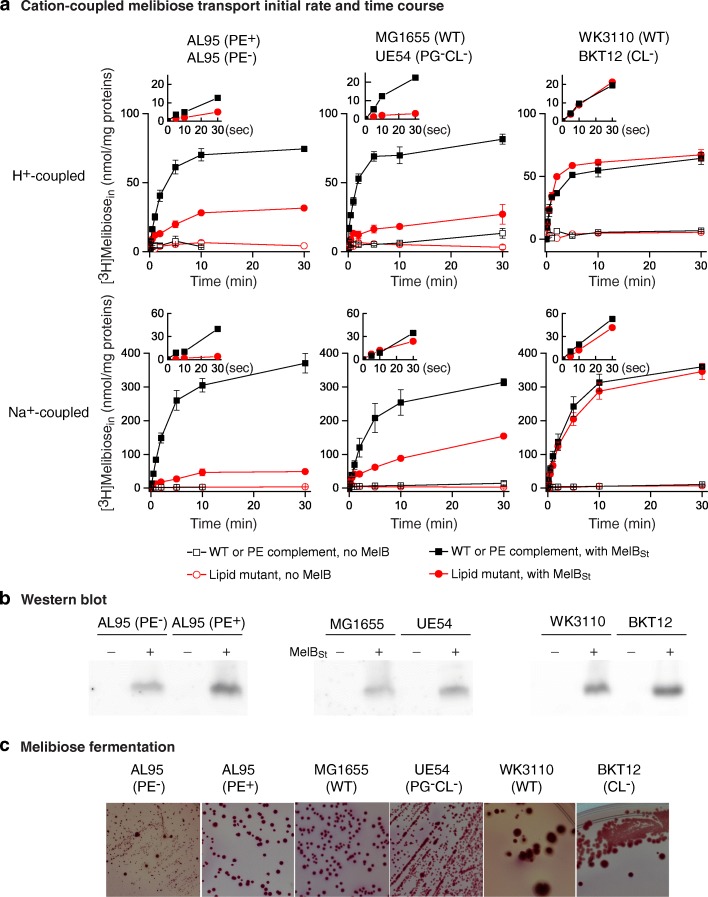
Effect of the major bacterial lipids on MelB_St_ protein expression and melibiose transport activities. **a** Melibiose transport with intact cells. Cells with varied lipid compositions without (open symbols) or with (filled symbols) MelB_St_-expressing vector pK95 ΔAH/MelB_St_/CHis_10_ were grown in LB as described in Methods. The melibiose transport assay in the absence or presence of 20 mM NaCl is described in the Methods. The intracellular melibiose was plotted against the incubation time. Inset, initial rate of transport within 30 s. Left column, strains AL95 (PE^−^) and AL95 with pDD72GM (PE^+^). Middle column, strains MG1655 (WT) and UE54 (PG^−^ CL^−^). Right column, strains WK3110 (WT) and BKT12 (CL^−^). Black curves, the WT or strain AL95 with pDD72GM; red curves, the lipid-deficient strains. Error bar, SEM; and the number of tests = 4–6. **b** Membrane expression. An aliquot of cells prepared for the transport assay in panels a-c were used to prepare the crude membrane fraction as described in the Methods. Membrane proteins of 20 μg from each sample were analyzed with SDS-15%PAGE, and MelB_St_ was detected by western blot using Penta⋅His HRP antibody. **c** Melibiose fermentation. Cells with varied lipid compositions transformed with pK95 ΔAH/MelB_St_/CHis_10_ were plated on the melibiose-containing MacConkey agar as described in Methods. Colonies on the MacConkey agar plates were grown at 37 °C, except for the strain AL95 with pDD72GM that was placed in a 30 °C incubator

The *E. coli* AL95 strain (*lacY*^−^) is a PE-deficient strain (Tables 1 and 2) [33]. A plasmid pDD72GM carrying the phosphatidylserine synthase-encoding *pssA* gene (the replication is temperature sensitive) was used for PE complementation (Tables 1 and 2) [33]. This pair of strains AL95 (PE^−^) and AL95 with pre-transformed pDD72GM plasmid (PE^+^) was transformed by an IPTG-independent constitutive expression plasmid pK95AH/MelB_St_/CHis_10_ encoding MelB_St_ with a 10xHis tag at the C terminus. Cells were grown in the presence of glucose to suppress the *melAB* operon. A [^3^H]melibiose transport assay with the PE^−^ strain showed that the initial rate and steady-state level of melibiose accumulation for both H^+^- and Na^+^-coupled transport modes are largely reduced (Fig. 2a, left column). MelB_St_ expression in the PE^−^ strain is about 80% of that in the PE^+^ strain (Fig. 2b), strongly indicating that PE is important for MelB_St_ transport activity.

**Table 1:** Strains and plasmids

	Genotype or description	Reference
*E. coli* strain		
AL95 (PE deficiency)	*pss93*::*kanR lacY*::*Tn9*	[33]
MG1655 (WT)	F^−^ lambda^−^ *ilvG*^−^ *rfb*^−^ 50 *rph*-1	[34]
UE54 (PG deficiency & CL deficiency)	MG1655 lpp-2 Δ*ara*714 *rcsF*::miniTn10cam Δ*pgsA*::FRT-Kan-FRT *(pgsA* encodes phosphatidylglycerol phosphate synthase)	[34]
WK3110 (WT)	F^−^ lambda^−^ IN(*rrn*D^−^*rrn*E)	[33]
BKT12 (CL deficiency)	WK3110 Δ*clsA*, Δ*clsB*, Δ*clsC*::KanR (cardiolipin synthases (Cls) catalyze the condensation of two PG molecules to one CL and one glycerol)	[35]
DW2	*melA*^+^ Δ*melB* Δ*lacZY*	[37]
Plasmid		
pK95 ΔAH/MelB_St_/CHis_10_	MelB_St_ with a C-terminal His_10_ tag (constitutive expression; ampicillin resistant).	[6, 37]
pDD72GM	*pssA*^+^ *genR* and pSC101 temperature-sensitive replicon (IPTG induction; chloramphenicol resistant).	[20]

[Note that *clsC* uses PG and PE to make CL plus ethanolamine]

**Table 2:** Lipid compositions in *E. coli* strains used in this study

Strain	PE	PG	CL	PA	Reference
UE54	90%	ND	ND	10% (with N-acy-PE)	[56]
AL95	ND	45%	50%	5%	[33]
WK3110	70–78%	12–15%	5.7–11%	1.5–2.1%	[35]
BKT12	70–79%	18–26%	ND	1.4–2.5%	[35]
AL95/pDD72GM	75%	20%	5–12%		[70]

*ND* not detectable

### Effect of anionic lipids PG or CL on melibiose active transport by MelB_St_

**Fig. 3:**
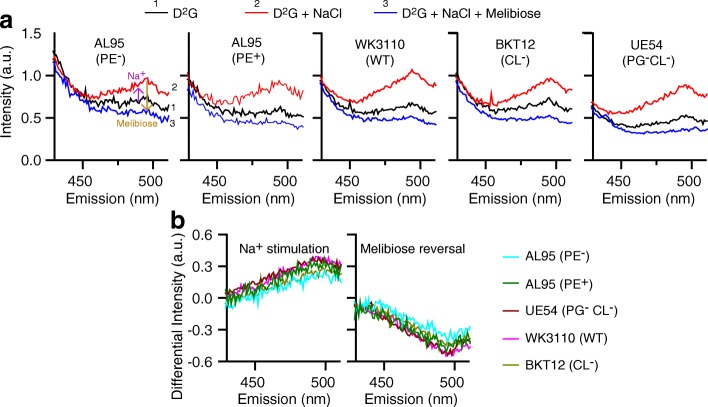
Determination of in situ binding of galactosides and Na^+^ with RSO membrane vesicles. A FRET assay of Trp→dansyl moiety of a fluorescent sugar substrate dansyl-galactopyranoside (D^2^G) was used for testing the binding of galactoside and the coupling of Na^+^ to MelB_St_ in varied lipid compositions as described in the Methods. **a** Trace 1, collected after mixing RSO vesicles (1 mg/ml) with 10 μL D^2^G; trace 2, collected after consecutive addition of 20 mM NaCl; trace 3, collected upon consecutive addition of melibiose at saturating concentration. **b** Differential FRET (_diff_FRET). Left panel, _diff_FRET spectra upon the addition of Na^+^ were calculated between the trace 2 and trace 1, reflecting Na^+^ binding. Right panel, _diff_FRET spectra upon the addition of melibiose were calculated between the trace 3 and trace 2, reflecting the galactoside binding

Strain UE54 was derived from the parent WT strain MG1655 with a single gene deletion of *pgsA* that encodes the phosphatidylglycerol phosphate synthase, lacking both PG and CL [34]. Since PG is the precursor of CL, the lack of CL results from the absence of PG. Notably, the content of PE in this strain is increased up to 90–95%, and there are 5–10% other anionic lipids to support cell viability (Tables 1 and 2). Strain BKT12 lacks CL, which was derived from the WT strain WK3110 by triple gene deletions on *clsABC* genes that encode three cardiolipin synthases [35]. All four strains have an intact *lacY* gene encoding LacY and an intact *melAB* operon encoding α-galactosidase and MelB_Ec_; both LacY and MelB_St_ also transport melibiose. It is known that the *melAB* operon is induced by the presence of its specific inducer melibiose, but not by IPTG [36], and glucose suppresses the activation of both *mel* and *lac* operons. To simplify the complexity, we again used the IPTG-independent, constitutive plasmid pK95AH/MelB_St_/CHis_10_, which allows us to test melibiose transport specifically mediated by a plasmid-encoded MelB_St_ [6, 37]. With Penta⋅His HRP antibody, the western blot shows a similar level of MelB_St_ expression (Additional file 1: Figure S1, upper panel), and LacY is not expressed under the growth conditions containing glucose (Additional file 1: Figure S1, lower panel**)**. In addition, the [^3^H]melibiose transport and Trp→D^2^G FRET assays (Figs. 2a and 3) also suggest that there is no expression of chromosomal MelB_Ec_. Thus, the phenotypes described in these studies reflect the transport catalyzed by the recombinant MelB_St_ encoded from the plasmid.

The transport time courses show that the CL- and PG-deficient strain UE54 exhibits a significantly reduced steady-state level of melibiose accumulation, with approximately 30% (H^+^-coupled) or 40% (Na^+^-coupled) of that from its parent WT strain MG1655 **(**Fig. 2a, middle column). The H^+^-coupled transport initial rate is even not detectable; however, the Na^+^-coupled transport initial rate is indistinguishable from the WT (Fig. 2a, inset). Notably, this effect is different from the PE effect (Fig. 2a). Interestingly, when MelB_St_ is expressed in the CL-deficient strain BKT12, in which PG is present, both of H^+^- and Na^+^-coupled melibiose transport is indistinguishable from its parent strain WK3110 (Fig. 2a, right column). Notably, the MelB_St_ protein expression in these mutant strains is not affected (Fig. 2b). Hence, our data demonstrate that PG plays important role in melibiose active transport activity.

### Melibiose fermentation

All the three pairs of lipid strains carrying the plasmid pK95AH/MelB_St_/CHis_10_ were also grown on MacConkey agar indicator plates containing 30 mM melibiose. Notably, in this medium, melibiose is the sole carbohydrate source for cell growth, and the rate of melibiose transport is the limiting step for melibiose utilization, which can be indicated by the red color development of the colonies due to acidification resulting from sugar utilization [14, 38, 39]. The degrees of acidification may reflect the melibiose down-hill transport activities. The intact *melAB* operon in all these strains can be induced by the presence of melibiose; thus, the resulting phenotypes should reflect the down-hill transport activity mediated by both endogenous MelB_Ec_ and recombinant MelB_St_. All these six strains ferment melibiose well, while strain AL95 PE^−^ grew slowly and formed many small colonies (Fig. 2c). The data show that lack of PG or PE exhibits little effect on melibiose downhill transport activities, while both lipid headgroups are important for melibiose uptake against the concentration gradient.

### Effect of lipid headgroups on the binding of Na^+^ and melibiose to MelB_St_ by FRET

To test the effect of lipid headgroups on the initial steps of transport in situ, a well-established Trp→D^2^G FRET assay was applied to detect the substrate binding, which is based on a fluorescent sugar substrate 2′-(N-dansyl)aminoalkyl-1-thio-β-D-galactopyranoside (D^2^G, dansyl-galactoside) [6, 40]. The right-side-out membrane (RSO) vesicles in the absence of Na^+^, which were prepared from the WT and lipid strains expressed MelB_St_ under the same growth condition as for the transport assay, were mixed with D^2^G at a concentration similar to its *K*_d_ value. Emission spectra were recorded between 430 and 510 nm (Fig. 3a). Emission peaks were detected with a maximum intensity around 495 nm (curve 1) from all samples. This is a signature for MelB_St_ because MelB_Ec_ should have another peak around 465 nm [6, 40]. The intensities are elevated (up-arrow) after adding 20 mM NaCl into the reaction mixture (curve 2) and decreased to a level below the first trace (down-arrow) when continually adding a saturating concentration of melibiose (curve 3). The increase in fluorescent intensity by Na^+^ is mainly due to the greater D^2^G binding affinity induced by Na^+^ [15], i.e., likely more D^2^G binding, and could be also partially from Na^+^-induced conformational changes of MelB_St_ [6, 40]. The reversal in fluorescent intensity reflects the displacement of bound D^2^G by the competitive binding of non-fluorescent melibiose. Previous studies have showed that this displacement is specific to the addition of MelB_St_ sugar substrates [6, 40].

MelB_St_ proteins in the varied lipid compositions (WT, PE-deficient, CL- & PG-deficient, or CL-deficient membrane) exhibit similar levels of Na^+^ stimulation and melibiose reversal of the D^2^G FRET (Fig. 3b), indicating that the MelB_St_ expressed in different lipid-deficient strains exhibits similar binding affinities for galactosides or Na^+^. This finding clearly demonstrates that the PE, PG, or CL headgroups are not important for the co-substrate binding with MelB_St_; thus, the initial steps of transport are not affected in the absence of PE and PG.

### Effect of lipid headgroups on MelB_St_ folding and stability by circular dichroism (CD) spectroscopy

An in situ test was carried out by incubating the RSO membrane vesicles carrying MelB_St_ prepared from the varied lipid strains at 45 °C for 90 min. After detergent solubilization using dodecyl-β-D-maltopyranoside (DDM) and ultracentrifugation to remove aggregations, the supernatants were analyzed by western blot. This in situ study shows that lack of CL alone, PG and CL, or PE, does not affect MelB_St_ resistance to heat treatment at 45 °C (Additional file 2: Figure S2).

**Fig. 4:**
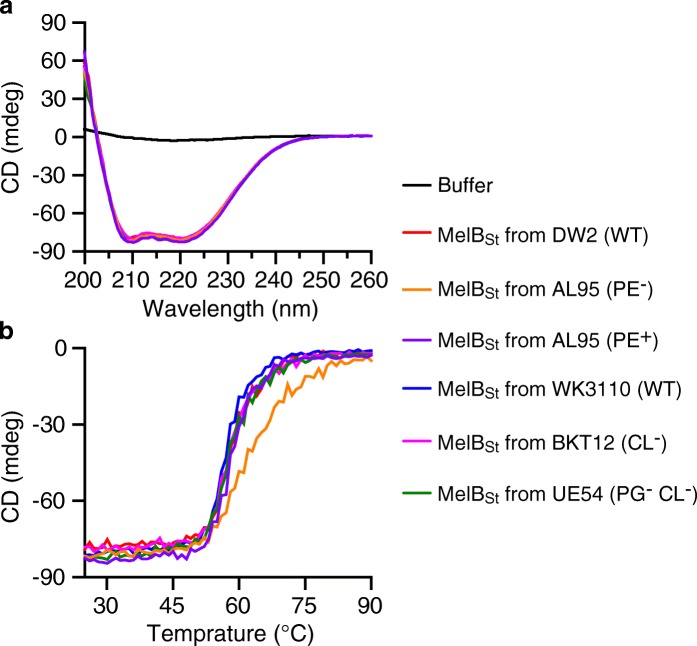
CD spectra and *T*_m_ determination. MelB_St_ was purified from varied lipid strains as described in Methods. **a** CD spectra. MelB_St_ at 10 μM of protein in a buffer containing 20 mM NaPi, 100 mM NaCl, 10% glycerol and 0.035% UDM was placed in 1 mm quartz cuvette in a temperature-controlled cell holder. The spectra were recorded between 200 and 260 nm and subtracted with corresponding buffer backgrounds. **b** The thermal denaturation profiles. The CD ellipticity changes was recorded at 210 nm each degree with temperature ramping 1 °C per minute

The CD spectroscopy was used to examine MelB_St_ protein folding and thermostability in vitro. MelB_St_ proteins were purified from the lipid-deficient strains and their parent strains including another *E. coli* WT strain DW2, which is routinely used for MelB structural and functional studies [2, 6, 15, 39]. Similar CD spectra are obtained with all of the MelB_St_ samples (Fig. 4a), showing that MelB_St_ mainly exhibits α-helical secondary structures as indicated by the two negative ellipticity peaks at 209 nm and 221 nm. The data are consistent with the 3-D crystal structure (Fig. 1) and also strongly indicate that MelB_St_ is correctly folded in these PE^−^, PG^−^CL^−^, or CL^−^ lipid strains.

Thermal-denaturation test for all of the samples were carried out at temperatures between 25 and 90 °C. CD spectra were recorded in intervals of 2 °C, and the ellipticity at 210 nm was separately monitored at each temperature, which was used to determine the *T*_m_ values. With MelB_St_ produced in DW2 strain, the content of the α-helical secondary structures, as detected at 210 nm, starts to rapidly decrease at 50 °C and completely disappeared at 75 °C, yielding a melting temperature (*T*_m_) value of 58 °C (Fig. 4b; Table 3). MelB_St_ samples produced from varied lipid-deficient strains exhibit comparable *T*_m_ values, clearly showing that the major phospholipid headgroups afford little or no effect on MelB_St_ protein folding and thermostability.

**Table 3:** MelB_St_ *T*_m_ determination

MelB_St_ from varied strains	Delipidation treatment	*T*_m_ (°C)
WT in UDM (DW2)	Before	58.01 ± 0.01^a^
	After	60.95 ± 2.53
WT in DDM (DW2)	Before	58.42
	After	60.01
PE^−^ (AL95)	Before	61.11 ± 0.62
	After	57.11
PE^+^ (AL95)	No treatment	58.52 ± 0.29
PG^−^ and CL^−^ (UE54)	Before	57.38 ± 0.26
	After	59.29
WT (WK3110)	No treatment	57.45 ± 0.45
CL^−^ (BKT12)	No treatment	58.29 ± 1.15

^a^SEM, standard error; number of tests is 2–3

### Identification of tightly bound anionic lipids with ssNMR spectra

To investigate lipid-MelB_St_ interactions, uniformly [U-^13^C^15^N]-labeled MelB_St_ was recombinantly produced in *E. coli* DW2 cells. After purification, the [U-^13^C,^15^N]-labeled MelB_St_ proteins were reconstituted into proteoliposomes using *E. coli* extract polar lipids at a protein to lipid ratio of 1:1.33 (mg:mg). The Trp→D^2^G FRET measurements with the proteoliposome samples were recorded by a time trace at an emission wavelength at 490 nm and an excitation wavelength at 290 nm (Additional file 3: Figure S3). Increased intensity was obtained after adding D^2^G, and reversed by addition of melibiose, but not by water, indicating that the reconstituted [U-^13^C,^15^N]-labeled MelB_St_ maintains the binding capability for both galactosides D^2^G and melibiose.

**Fig. 5:**
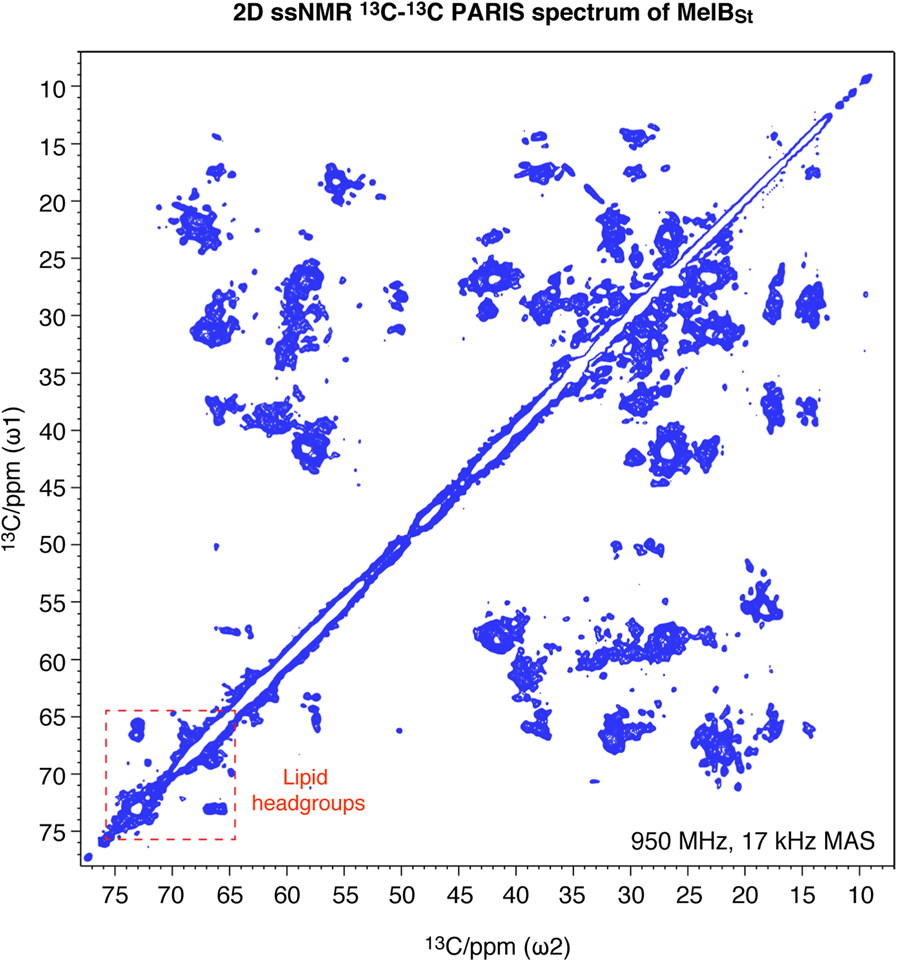
A 2D ^13^C-^13^C PARIS ssNMR spectrum of proteoliposomes containing [^13^C,^15^N]-labeled MelB_St_, measured at 950 MHz magnetic field using 17 kHz MAS with a mixing time of 40 ms at 265 K. The lipid-headgroup signature region is highlighted by a red-dashed box

**Fig. 6:**
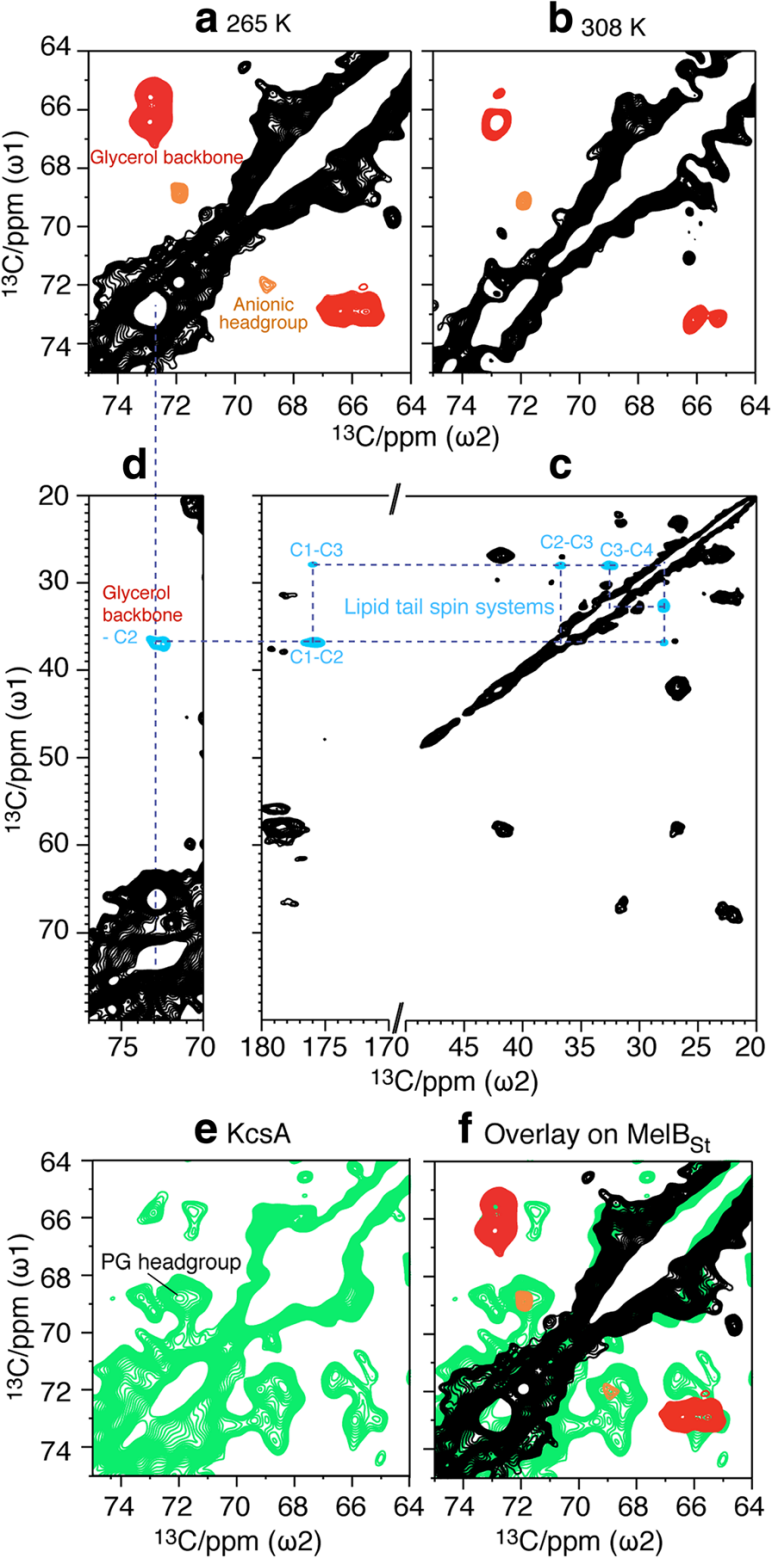
ssNMR characterization of phospholipids co-purified with MelB_St_. **a**–**c** Cut-outs from 2D PARIS ^13^C-^13^C PARIS spin diffusion ssNMR spectra of MelB_St_, which were measured at 265 K (panels **a** or **c**) or 308 K (panel **b**) sample temperature, and with 40 ms mixing time. The correlations of glycerol backbones (red in panel **a** and **b**), anionic headgroups (orange in panel **a** and **b**), and the tail carbons (cyan in panel c) of the endogenous lipids, are illustrated. **d** A 2D PARIS-xy ^13^C-^13^C ssNMR spectrum with a mixing time of 160 ms shows clear correlations between lipid glycerol backbone and lipid tail carbons. Illustrative carbon-carbon connectivities are highlighted with blue-dashed lines. **e** Cut-out of the lipid headgroup region of a 2D ^13^C-^13^C PARIS ssNMR spectrum of reconstituted, membrane-embedded K^+^ channel KcsA, which was purified from *E. coli* cells. The correlations (69–72 ^13^C ppm) from the headgroups of endogenous, co-purified anionic lipids, which are assumed to be PG headgroups [29, 47], are indicated. **f** Overlay of a 2D PARIS spectrum of MelB_St_ on KcsA

A dipolar-based two-dimensional (2D) ^13^C-^13^C PARIS [41, 42] spin diffusion experiment with a short ^13^C-^13^C mixing time of 40 ms was carried out at 950 MHz (^1^H-frequency) magnetic field using 17 kHz magic angle spinning (MAS) frequency and a real temperature of approximately 265 K. A high-quality spectrum was obtained, featuring many resolved cross-peaks (Fig. 5) as narrow as 0.4–0.5 ^13^C ppm (95–120 Hz at 950 MHz). The resulting signal pattern is in good agreement (Additional file 4: Figure S4a) with the predictions [43] of chemical shifts calculated from the MelB_St_ X-ray structure [2] (PBD ID, 4 M64). Interestingly, strong ^13^C-^13^C cross-peaks around 65–75 ^13^C ppm are observed, which is a typical fingerprint of the headgroup and the glycerol backbone of phospholipids [29]. These correlations are clearly not protein signals (Figs. 5 and 6a) and must originate from endogenous ^13^C-labeled lipids, which were co-purified with MelB_St_ from the *E. coli* DW2 inner membrane. The signals from the beginning of the lipid alkyl tails (C1–4) can also be clearly identified. Lipid carbons of the beginning of the tails (around 30–40 ^13^C ppm), involving the carbonyl C1 carbon at 176 ^13^C ppm represent a spin system that also does not exist in proteins (Fig. 6c, in cyan), and the observed chemical shifts agree well with published assignments for lipid tails [44]. These lipid signals exhibit stronger intensities than most protein signals in the 2D ^13^C-^13^C PARIS spectrum (Fig. 6c, in cyan). Moreover, using a PARIS-xy experiment [45] at 950 MHz and 17 kHz MAS that specifically enhances the transfer between spectral regions separated by ~ 30–50 ^13^C ppm and a longer ^13^C-^13^C spin diffusion time of 160 ms, we could establish a clear correlation between the glycerol backbone and the lipid tail C2 carbon **(**Fig. 6d). This unambiguously demonstrates the presence of rigid acyl tails of endogenous lipids in the spectrum. Therefore, the entire lipid molecule must be rigid on the micro- to millisecond timescale, which is the time-scale of the relevant dipolar couplings that drive spin diffusion. Furthermore, these lipid signals remain strong even at elevated temperature of 308 K (Fig. 6b), which also supports that the detected lipids behave differently from bulk lipids. Notably, lipid carbons further down the tail are also likely present in the spectra; however, these carbons exhibit the same chemical shifts and their correlations overlap with the spectral diagonal, so that they cannot be assigned in the spin diffusion spectrum. Moreover, specific contacts between MelB_St_ and the lipid tail/glycerol-backbone are supported by a 2D ssNMR PARIS-xy spectrum with a very long ^13^C-^13^C mixing time of 750 ms (Additional file 4: Figure S4b, blue). The cross-peaks highlighted by a magenta box with signals between ~ 63–57 ^13^C ppm are consistent with specific protein-lipid contacts.

To identify the species of the tightly bound lipids, the lipid signature region was further analyzed. Two correlations at 65.8–73.0 and 66.6–73.0 ^13^C ppm can be unambiguously assigned to the glycerol backbone of the co-purified lipids (Fig. 6b, in red) [46]. There are also weaker but well-resolved and symmetric correlations at 69.0–72.1 ^13^C ppm (Fig. 6a, b, in orange), which probably stem from the headgroups of co-purified, ^13^C-labeled anionic lipids. The ^13^C signals for the headgroups of anionic lipids have been reported around 69–72 ^13^C ppm [29, 44], while the ^13^C signals for the zwitterionic PE headgroup would occur at much lower ^13^C ppm values (55–67 ^13^C ppm**)** [46]. Further tests with 1,2-dioleoylphosphatidylglycerol (DOPG) liposomes and DOPE:DOPG liposomes (9:1 ratio) show that PG headgroup signal resonates between 69 and 72 ^13^C ppm (Additional file 5: Figure S5), while PE headgroup does not feature ^13^C signals at this range. Moreover, the headgroup signals overlay well with the endogenous anionic PG that were co-purified with the K^+^ channel KcsA (Fig. 6e, f) [29, 47]. Hence, MelB_St_ with tightly bound PG could be established with ssNMR spectroscopy.

### Determination of lipids that are tightly bound to MelB_St_ by mass spectrometry and thin layer chromatography (TLC)

**Fig. 7:**
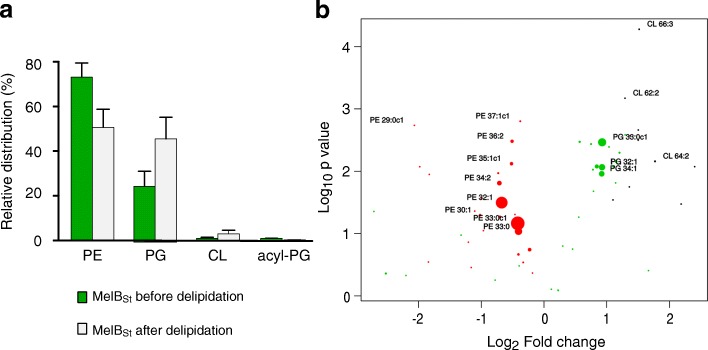
Lipids associated with MelB_St_. Lipids associated with purified MelB_St_ protein before and after a delipidation treatment were analyzed by HPLC/MS. **a** Molar contributions of lipids were calculated using response factors obtained from the analysis of known quantities of authentic lipid standards. For acyl-PG, the PG identical response factors were assumed because of lack of a standard for acy-PG. **b** Volcano plot. The statistics *P* value (log_10_) is plotted against the fold-change (Log_2_). Dot sizes for all species correspond to their relative contribution to the lipid pool associated with MelB_St_ protein. Red, PE; green, PG; black, CL

MelB_St_ protein samples purified from *E. coli* DW2 cells, which is the same strain used for U-^13^C^15^N labeling, were subjected to lipid analyses by mass spectrometry and TLC. Abundant PE, PG, and trace of CL co-purified with MelB_St_ were detected. PE is the dominant species counting for approximate 70% of the total lipids, PG counts for approximately 25% PG, and the rest corresponds to other minor species including CL and acyl PG (Fig. 7a; Additional file 6: Figures S6a and Additional file 7: Figure S7). The majority of lipids contain acyl chain lengths of 16–17 carbons (Additional file 8: Figure S8a). Dual phosphorus (P_*i*_) and protein concentration assays were carried out showing that the ratio of lipids to MelB_St_ is 20.62 ± 1.07:1 mol/mol if we ignore the trace amount of CL that contains two P_*i*_ (Table 4).

**Table 4:** Lipids co-purified with MelB_St_

MelB_St_ from WT DW2 cells	PE	PG	PL:MelB_St_ (mol:mol)
Before delipidation	70%	25%	20.62±1.07: 1^a^
After delipidation	50%	50%	2.95±0.13: 1

^a^SEM, standard error; number of tests is 3

To analyze the tightly bound lipids, three preparations of MelB_St_ were subjected to detergent washing to remove co-purified lower-affinity lipids. The delipidated samples exhibit a largely decreased lipids to protein ratio of 2.95 ± 0.13:1 (mol/mol), and TLC and mass spectrometry consistently show a decreased PE:PG ratio of approximate 50:50 (Fig. 7a; Additional file 7: Figure S7; Table 4). Notably, change in the lipid chain lengths and lipid unsaturation before and after delipidation treatment were also detected (Additional file 8: Figure S8). The volcano plot of statistics *P* value versus fold-change clearly reveals that PG is enriched in the delipidated MelB_St_ at a cost of PE (Fig. 7b). Together, the results strongly indicate that there are at least one tightly bound PE and one tightly bound PG in MelB_St_ samples. The presence of tightly bound PG is also revealed by ssNMR spectra.

**Fig. 8:**
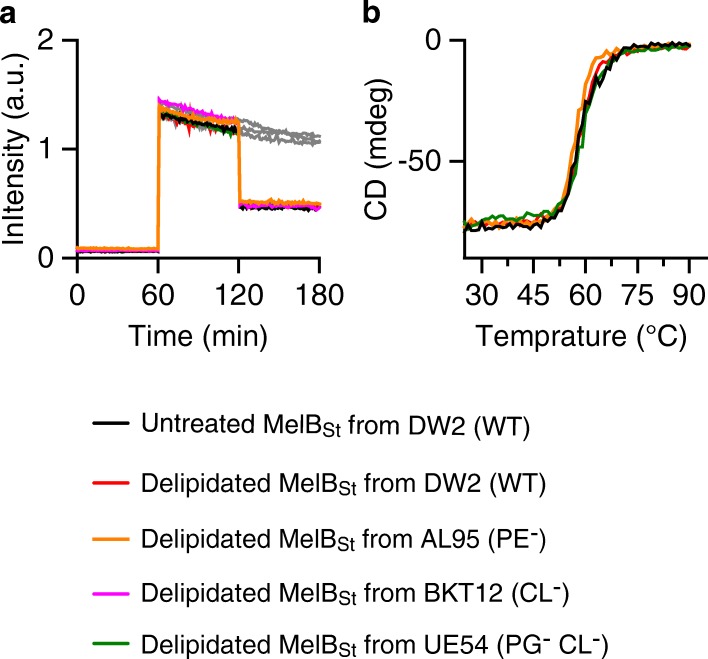
Effect of delipidation on MelB_St_ binding and stability. MelB proteins purified from varied lipids strains were subjected to the delipidation treatment as described in Methods. **a** Galactoside binding by the Trp→D^2^G FRET assay. The delipidated MelB_St_ at 1 μM was used to test binding of galactosides by Trp→D^2^G FRET assay based on a time trace. **b** Determination of *T*_m_ value by CD spectra. The delipidated MelB_St_ at 10 μM in a buffer containing 20 mM NaPi, 100 mM NaCl, 10% glycerol and 0.01% DDM are used for thermal denaturation test by monitoring CD ellipticity changes at 210 nm between temperatures 25–90 °C

Remarkably, the delipidated MelB_St_ with few tightly bound lipids exhibits the galactoside-binding capability and *T*_m_ value comparable to MelB_St_ in the absence of delipidation treatment (Fig. 8; Table 3). Furthermore, even the MelB_St_ purified from the PE^−^ strain (AL95) or PG^−^CL^−^ strain (UE54) shows a similar galactoside-binding affinity and *T*_m_ values, strongly indicating that MelB_St_ stability only requires the presence of a few tightly bound lipid acyl chains and is independent of the lipid head groups.

## Discussion

X-ray crystallography reveals that most transmembrane helices of MelB_St_ are heavily distorted with tilts and kinks ([2]; Fig. 1). This structural information raises interesting questions how the surrounding lipids interact with MelB_St_, and how these lipids support and adapt MelB protein conformational changes and structural rearrangements during transport. To address these important questions, in this study, we have utilized an integrated approach and characterized the modulating effects of phospholipids on MelB_St_ structure and function. Mass spectrometry, phosphorus assay, and thin layer chromatography reveal that the major phospholipids co-purified with MelB_St_ are zwitterionic PE (70%) and anionic PG (25%) at an estimated lipid to protein ratio of 21:1 (mole/mole) (Table 4). After extensively removing lipids by detergent washing, this ratio decreases to 3:1 (mole/mole) with a PE:PG ratio of approximately 1:1. More PE molecules were removed by detergents; as the result, PG is enriched after delipidation. Our data also demonstrate that at least one non-exchangeable PE and one non-exchangeable PG tightly bind to MelB_St_.

Solid-state NMR not only enables to probe membrane protein structure and dynamics in native-like membranes but also enables the investigation of lipid-protein interactions [29, 48–53], including the individual interactions of hydrophobic tail, glycerol backbone, and headgroup, as we demonstrate here. MelB_St_, as a large (52 kDa) and predominately (> 70%) α-helical transmembrane protein [2], presents a considerable challenge for ssNMR studies in terms of both sensitivity and resolution. Here, we present high-quality ssNMR spectra (Figs. 5 and 6), which allow us to clearly identify the non-exchangeable PG headgroups and lipid acyl chains in MelB_St_, which agrees with the results obtained from mass spectrometry and TLC chromatography. The ssNMR spectra show no signals for PE headgroups, while PE is clearly identified by mass spectrometry and TLC chromatography. Interestingly, the ssNMR signals of the PG headgroup are approximately three-times weaker than the signals of the lipid glycerol backbone and the acyl chains. This raises the possibility that the backbone of the non-exchangeable PE may also contribute to this stronger signal; the PE headgroups may be dynamic so that it is not detectable because the signal intensity in our dipolar-based spectra decreases with molecular mobility increase. It is also possible that increased dynamics of the bound PG headgroup may partially contribute to the relatively weaker headgroup signals. In either case, the interactions of these endogenous lipids with MelB_St_ are mainly based through their lipid acyl chains and glycerol backbone. Notably, the ssNMR signals on the PG headgroup, glycerol backbone, and the carboxylic part of acyl groups are the full invariant part of all PG species. Moreover, the lipidomic-based analysis of the lipid species and the lipid class, as an independent experimental technique, shows no shift in lipid species pattern for delipidated samples, which agrees with the PG-protein interactions. Interestingly, the type of lipid-protein interactions described here is different for that with the channel protein KcsA, for which only the headgroups, but not the tails, of co-purified lipids are detectable by ssNMR at ambient temperature [29].

To determine the role of the headgroups and the acyl chains for MelB stability, in situ and in vitro thermal-denaturation tests were carried out. MelB_St_ in varied membrane lipids compositions is resistant to a 45 °C treatment, and MelB_St_ purified in detergent UDM from WT strain or the mutant strains (PE^−^, CL^−^, and PG^−^CL^−^ strains) exhibits comparable *T*_m_ values (Table 3). Intriguingly, the *T*_m_ value for the delipidated MelB_St_ produced from PE^−^ and PG^−^CL^−^ strains (Table 3) is also unchanged, which strongly supports the notion that the headgroups play little or no role for the MelB_St_ stability, and even few tightly bound lipid tails can maintain the thermostability of MelB_St_. Rigid lipid tails are observed by ssNMR, indicating strong hydrophobic interactions (Fig. 6; Additional file 4: Figure S4b). This type of interaction may have important biological roles in transport processes. This transporter protein must adopt several largely different conformations; thus, it has to be structurally labile, allowing conformational transitions to cycle among several kinetic states. The use of nonspecific interactions through a large area of the lipid acyl chains and glycerol backbone can function as a firm and flexible “grip” that will enable the lipids to follow the protein conformational changes more easily, supporting the protein folding at different conformations.

To investigate the effect of headgroups on the MelB binding and transport activity, we performed detailed biochemical analyses. Because of the presence of non-exchangeable lipids, a genetic approach to alter the cell membrane lipid headgroup compositions was applied (Table 2; reviewed in references [54, 55]). Notably, PG is the precursor of CL, so it is challenging to have a strain that contains CL but lacks PG. In addition, the strain that lacks both CL and PG (UE54) is significantly enriched in other anionic lipids (PA and *N*-acyl-PE) that compensate for the loss of anionic PG and CL and support the cell viability [56]. With these lipid-deficient strains, the expression of MelB_St_ and binding for melibiose and Na^+^ are not much affected (Figs. 2 and 3), while the expression level with PE-deficient strain is reduced. The results strongly argue for the conclusions that none of the lipid headgroups is involved in the galactoside binding nor the cation H^+^ or Na^+^ binding.

These lipid-deficient strains behave differently with regard to the active transport against melibiose concentration gradient. In the PE-deficient strain, both of the initial rate and level of steady-state of the transport are dramatically inhibited, regardless of the H^+^- or Na^+^-coupled transport modes. It has been reported that the membrane vesicles prepared from PE-deficient cells can maintain cell electrochemical H^+^ gradient [18, 57]. Thus, the inhibition on the electrochemical H^+^ gradient-driven transport activity supports the notion that PE plays important roles to enable MelB_St_ to catalyze active melibiose uptake while the specific steps have not been identified yet. This PE effect on MelB is quite different from its dramatic effect on the overall structure of LacY [20, 22, 25] or GabP [27].

When both PG and CL are lacking, the active melibiose transport is also largely inhibited, particularly with the H^+^-coupled transport. Interestingly, no effect on the initial rate is observed when the transport is coupled to Na^+^ electrochemical gradient; however, when coupled to H^+^ electrochemical gradient, the transport initial rate is largely inhibited. The CL-deficient strain behaves like the WT, which strongly indicates that CL is not required and the transport inhibition observed in the CL- and PG-deficient strain is solely caused by the lack of PG. PG headgroup is observed by ssNMR, which is likely the only type of lipid headgroup that strongly interacts with MelB_St_ and plays important role(s) in the cation-coupled melibiose transport. The negative charged PG headgroups could dynamically interact with the positively charged sidechain(s) presenting in the membrane-aqueous interface of MelB_St_ and modulate the protein conformational changes. Overall, as clearly shown by mass spectrometry and TLC, PE and PG are both tightly bound to MelB_St_, and lack of either species entails functional consequences.

## Conclusions

In summary, differential roles of lipid headgroups and acyl chains are identified with MelB_St_. The lipid headgroups of PE and PG are critically involved in the cation-coupled melibiose uptake. However, specific interactions with lipid headgroups play little or no role in MelB_St_ folding, substrate binding, nor melibiose downhill transport. With regard to the folding and stability, MelB_St_ relies on only few tightly bound lipids acyl chains.

## Methods

### Materials

The 2′-(N-dansyl)aminoalkyl-1-thio-β-D-galactopyranoside (D^2^G) was obtained from Drs. H. Ronald Kaback and Gérard Leblanc. [1-^3^H]Melibiose was custom-synthesized (PerkinElmer). Undecyl-β-D-maltopyranoside (UDM), dodecyl-β-D-maltopyranoside (DDM), and octyl-β-D-glucoside (OG) were purchased from Anatrace. MacConkey agar media (lactose free) was purchased from Difco. [U-^13^C]glucose and [^15^N]NH_4_Cl were purchased from Cortectnet*. E. coli* lipids (Extract Polar) was purchased from Avanti Polar lipids, Inc. All other materials were reagent grade and obtained from commercial sources.

### Plasmids and strains

All bacterial strains and plasmids used in this study, their sources, and references, are listed in Table 1.

### Cell growth for functional assays

LB media were used for cell growth at 30 °C or 37 °C. For the growth of AL95 strain, 50 mM MgCl_2_ was supplemented [18]. For the strain AL95 carrying the temperature-sensitive plasmid pDD72GM, the cells were grown at 30 °C. Kanamycin at 12.5 mg/L was used for maintaining BKT12 genotype. Ampicillin at 100 mg/L was used for maintaining the plasmids pK95 ΔAH/MelB_St_/CHis_10_, and chloramphenicol at 30 mg/L was used for maintaining the plasmid pDD72GM.

### Isotope labeling, membrane preparation, and protein purification

DW2 cells containing the plasmid pK95 ΔAH/MelB_St_/CHis_10_ grown in 50-mL M9 minimal media overnight at 37 °C were inoculated into 1-L M9 media containing 0.2% [U-^13^C]glucose, 0.075% [^15^N]NH_4_Cl, and shaken at 37 °C for 16 h. This 1-L overnight culture was inoculated to a 9-L M9 media containing 0.2% [U-^13^C]glucose, 0.075% [^15^N]NH_4_Cl, and grew in 30 °C for 17 h to *A*_600_ = 1.6. About 38 g of wet cell pellets were collected.

Preparation of membrane samples by passing through Emulsiflex twice to break the cells and ultracentrifugation to collect the membranes were carried out as described previously [2]. MelB_St_ protein purification using 1.5% UDM to solubilize the membrane samples at a protein concentration of 10 mg/ml was also carried out as described [2, 15, 58]. Purified MelB_St_ was dialyzed against a buffer containing 20 mM Tris-HCl, pH 7.5, 100 mM NaCl, 10% glycerol, and 0.035% UDM in the absence of melibiose, and yielded 35 mg of highly pure [U-^13^C,^15^N]-labeled MelB_St_ protein sample from 10 L for reconstitution. MelB_St_ purification from varied strains were carried out using the same protocol as described above [2, 15, 58]. Crude membrane preparations were carried out as described [14, 36].

### MelB_St_ reconstitution by a dilution method

The reconstitution into proteoliposomes was carried with *E. coli* Extract Polar (Avanti) at a ratio of 1:1.33 (mg:mg). Briefly, 40 mg of the lipids dissolved in 1.2% OG was mixed with 30 mg of the [U-^13^C,^15^N]-labeled MelB_St_ samples in UDM. After a 30-min incubation at room temperature, the mixture was subjected to a 74-fold dilution by adding buffer containing 20 mM NaP_i_, pH 7.5, and 150 mM NaCl, and incubated for another 30 min with stirring before ultracentrifugation at 47,000 rpm on a Beckman rotor 70 Ti at 4 °C for 2 h. The pellets were re-suspended in the same buffer and subjected to three cycles of freeze-thaw-sonication. The sonication was carried out in an ice-cold bath sonicator (Branson 2510), 5 s for three times. The samples (5-mL) were washed once with 20 ml of the same buffer and concentrated to a protein concentration of 44 mg/ml by ultracentrifugation under same conditions. About 27 mg MelB_St_ proteoliposomes was obtained.

### Trp→D^2^G FRET assay

The Trp→D^2^G FRET assays [6, 40] were carried out in a 3-mm quartz cuvette (Hitachi F-7000 Fluorescence Spectrophotomer or AMINCO-Bowman Series 2 Spectrometer). When using time traces, the fluorescence intensity changes were recorded at an emission wavelength of 490 nm and an excitation wavelength of 290 nm before and after adding D^2^G and followed by melibiose. The purified MelB_St_ or the MelB_St_-proteoliposomes sample in 20 mM NaP_i_, pH 7.5, and 150 mM NaCl at 0.5 μM of protein concentration were as mixed with 10 μM D^2^G. After recording for 1 min, melibiose at a saturating concentration or equal volume of water were added. For the measurements with RSO vesicles at 1 mg/ml of protein concentration in 100 mM KP_i_ (pH 7.5) buffer, the emission spectra were recorded between 430 and 510 nm at an excitation wavelength of 290 nm at each of the followed conditions: (1) the samples were mixed with 10 μM D^2^G, and (2) consecutively added with 20 mM NaCl (testing Na^+^ stimulation) and (3) finally added with the melibiose at a saturating concentration (testing melibiose reversal).

### Solid-state NMR spectroscopy

Dipolar-based 2D PARIS [41, 42] (*N* = 0.5) and PARIS-xy [45] (N = 0.5, m = 1) ^13^C-^13^C experiments on membrane-embedded MelB_St_ were performed at 17 kHz magic angle spinning (MAS) frequency and 950 MHz (^1^H-frequency) static magnetic field (Bruker Biospin) at temperatures of approximately 265 K and 308 K. A recoupling amplitude of 10 kHz was applied for a total mixing time of 40 ms and 160 ms in the PARIS experiments and the PARIS-xy experiment, respectively. For each experiment, the phase of recoupling pulses was inverted after half a rotor period (*N* = 0.5). The 2D ^13^C-^13^C PARIS spectrum with membrane-embedded KcsA was performed at 700 MHz (^1^H-frequency) using 13 kHz MAS and a spin diffusion time of 150 ms.

### Melibiose transport assay

Melibiose transport activities in *E. coli* strains were accessed by [^3^H]melibiose flux assay as described previously [6, 39]. Cells in 100 mM KPi, pH 7.5, and 10 mM MgSO4 were adjusted to *A*_420_ = 10, and 50 μL aliquots were used to mix with [^3^H]melibiose at 0.4 mM (specific activity, 10 mCi/mmol) in the absence (H^+^-coupled transport) or presence of 20 mM NaCl (N^+^a-coupled transport). The intracellular amount of [^3^H]melibiose at a given time-point was collected by a fast filtration and measured by a scintillation counter (Beckman LS6500).

### Melibiose fermentation

Cells were transformed with pK95 ΔAH/MelB_St_/CHis_10_ and plated on the MacConkey agar supplemented with 30 mM melibiose [38, 59]. MacConkey media contains 85.6 mM NaCl, with supplement of 50 mM MgCl_2_ for the strains AL95 (PE^−^) and AL95 with pDD72GM. All plates were incubated at 37 °C except the strain AL95 with pDD72GM was plated in a 30 °C incubator. Pictures were taken after 1–2 days, except for the picture of AL95 (PE^−^) cells that was taken after 5 days.

### Preparation of right-side-out (RSO) membrane vesicles

The *E. coli* strains carrying the plasmid pK95 ΔAH/MelB_St_/CHis_10_MelB_St_ were grown in LB media supplement with 0.5% glycerol at 30 °C as described previously [6]. With the AL95 strains, 50 mM MgCl_2_ was added into the LB media. The RSO membrane vesicles were prepared by osmotic lysis as described previously [6, 60, 61], re-suspended with 100 mM KP_i_ (pH 7.5) and 10 mM MgSO_4_ at a protein concentration of ~ 20 mg/ml, and then stored at − 80 °C.

### MelB_St_ thermostability assay in situ

RSO membrane vesicles containing MelB_St_ at a protein concentration of 10 mg/mL in the presence of 20 mM NaPi, pH 7.5, 200 mM NaCl, 10% glycerol, 20 mM melibiose were incubated at 45 °C for 90 min, then put on ice and extracted with 1.5% DDM. The DDM-extracted solutions were subjected to ultracentrifugation at 355,590*g* in a Beckman Optima™ MAX Ultracentrifuge using a TLA-100 rotor for 30 min at 4 °C. To analyze the amount of MelB_St_ in the supernatant fractions, the RSO vesicles at 20 μg and equal volume of treated samples were analyzed by SDS-15% PAGE, and MelB_St_ signal was detected by western blotting with a Penta-His-HRP antibody (Qiagen).

### Delipidation of MelB_St_

Detergent washing was used to remove lipids loosely bound to membrane proteins [62]. The MelB_St_ protein at a concentration of 5 mg/ml in 20 mM Tris-HCl, 100 mM NaCl, 10% glycerol, and 0.035% UDM was incubated with 2% DDM for 16 h at 4 °C and loaded onto a column containing Talon resins. After being washed with buffer containing 2% DDM, MelB_St_ was eluted in a buffer containing 0.01% DDM and 0.2 M imidazole, dialyzed against 20 mM Tris-HCl, 100 mM NaCl, 10% glycerol, and 0.01% DDM, and concentrated to about 15 mg/ml.

### Inorganic phosphorus (P_i_) assay

The content of inorganic P_i_ was used to estimate the lipid content [62]. Paired MelB_St_ protein samples at 20 μg (before) and 50 μg (after delipidation) were subjected to phosphorus extraction and estimation using malachite green as described [63]. Briefly, phosphorus was extracted by adding 0.2 mL concentrated perchloric acid and heated at about 180 °C for 30 min, diluted with 0.8-mL water, and mixed with 1 mL of freshly prepared mixture containing 0.3% malachite green, 1.05% ammonium molybdate, and 0.045% Tween 20. Phosphorus standards in the concentration range of 0–0.6 μg were used. After being incubated at a room temperature for 1 h, the color development at 620 nm was measured by a UV spectrometer.

### CD spectroscopy

The CD measurements were carried out using Jasco J-815 spectrometer equipped with a peltier MPTC-490S temperature-controlled cell holder unit. A 200-μL sample of MelB_St_ at a concentration of 10 μM in a buffer containing 20 mM NaPi, 100 mM NaCl, 10% glycerol and 0.035% UDM (or 0.01% DDM) were placed in 1 mm quartz cuvette on the temperature-controlled cell holder. CD spectra were collected by using Jasco Spectra measurement version 2 software for a wavelength range of 200–260 nm with a data pitch of 0.1 nm using a band width of 1 nm and scanning speed of 100 nm/min. Each spectrum was corrected by subtraction with corresponding buffer background.

The thermal denaturation was monitored at 210 nm, and the temperature ramps 1 °C per minute. The melting temperature (*T*_m_) values were determined by fitting the data to the Jasco Thermal denaturation multi analysis module.

**TLC**. Lipids from MelB_St_ protein samples at 100 μg (before) or 400 μg (after delipidation) were extracted with 150 μl of CHCl_3_:MeOH (2:1, *v*/*v*), and further mixed with 150 μL of water and 150 μL chloroform. The lipid extracts in chloroform phase were collected after centrifugation at 3000*g* for 5, further dried by a SpeedVac Concentrator, and analyzed by TLC on a pre-coated Silica 60 plate (Merck, Darmstadt, Germany) using an alkaline solvent system [CHCl_3_:MeOH: 28% NH_4_OH:H_2_O (45:35:1.6:8, *v*/*v*/*v*/*v*)] [64]. The primuline solution at a concentration of 0.0005% was used for visualization.

### Liquid chromatography and mass spectrometry of lipids

Lipids were extracted from MelB_St_ protein samples before and after delipidation treatment using the method of Bligh and Dyer [65]. Chromatography of 10 μL of the supernatant was performed on a hydrophilic interaction liquid chromatography (HILIC) column (2.6 μm HILIC 100 Å, 50 × 4.6 mm, Phenomenex, Torrance, CA), by elution with a gradient from ACN/Acetone (9:1, *v*/*v*) to ACN/H2O (7:3, *v*/*v*, containing 10 mM ammonium formate), both with 0.1% formic acid, at a flow rate of 1 mL/min. The column outlet of the LC was connected to a heated electrospray ionization (hESI) source of a Fusion mass spectrometer (ThermoFisher Scientific, Waltham, MA). Full spectra were collected from m/z 400 to 1600 at a resolution of 120.000. Parallel data dependent MS2 was done in the linear ion trap at 30% HCD collision energy. Data were converted to mzML format and analyzed using XCMS version 1.52.0 [66] running under R version 3.4.3 (R Development Core Team: A language and environment for statistical computing, 2016. URL http://www.R-project.org).

### Protein concentration and visualization techniques

Protein concentration was assayed by a Micro BCA kit (Thermo Scientific). Plasmid-borne MelB_St_ expression was analyzed on SDS-15%PAGE, and MelB_St_ signal was detected by western blot with a Penta-His-horseradish peroxidase (HRP) antibody (Qiagen, Cat No./ID: 34460). Expression of chromosomally encoded LacY was evaluated with a site-directed polyclonal antibody against the C terminus of LacY [67, 68] (provided by H. Ronald Kaback) and HPR-conjugated protein A. The chemiluminescent signals were imaged by the ImageQuant LAS 4000 Biomolecular Imager (GE Healthcare Life Science).

## Additional files

Additional file 1: **Figure S1.** Protein expression. Cells were grown in LB media containing 10 mM glucose at 30 °C for 5 h, and cell membranes were prepared. 20 μg of total membrane proteins were analyzed by SDS-15%PAGE and western blot. (a). MelB_St_ expression was detected by Penta⋅His HRP antibody. (b). An anti-C terminal LacY antibody was used to detect LacY expression. (PDF 864 kb)

Additional file 2: **Figure S2.** MelB_St_ stability test in situ. RSO vesicles prepared from MelB_St_-expressing cells with different lipid compositions (sample S1) were incubated at 45 °C for 90 min, and then solubilized with detergent DDM (sample S2). After separation by ultracentrifugation, the soluble MelB_St_ retaining in the supernatant (sample S3) was analyzed by SDS-15% PAGE and western blot using Penta⋅His HRP antibody. (PDF 135 kb)

Additional file 3: **Figure S3.** Galactoside binding. A Trp→ D^2^G FRET assay was used to detect the binding of the [^13^C, ^15^N]-labeled MelB_St_ after reconstituted into proteoliposomes as described in the Methods. On the time trace set at an excitation wavelength of 290 nm and emission wavelength of 490 nm, D^2^G at 10 μM was added into the MelB_St_ liposome samples at 60-s time point, and melibiose at a saturation concentration or equal volume of water was further added into the solution at 120-s time point. (PDF 701 kb)

Additional file 4: **Figure S4** (a) Overlay of the 2D ^13^C-^13^C PARIS spectrum with predictions derived from the 3D X-ray crystal structure of MelB_St_. The 2D ^13^C-^13^C PARIS spectrum from Fig. 5 of the main text is superimposed with FANDAS [43] chemical shift predictions [69] derived from the MelB_St_ X-ray structure [PDB access ID, 4 M64]. Globally, the ssNMR signals match very well to the predictions. The headgroup signals of the co-purified lipids have no corresponding predictions from the protein. No lipid was resolved in the X-ray structure. (b) Specific contacts between MelB_St_ and lipid tail/glycerol-backbone. A 2D ssNMR PARIS-xy spectrum with a very long ^13^C-^13^C mixing time of 750 mx was measured at 250 K. The cross-peaks highlighted by magenta boxes are consistent with specific protein-lipid contacts. The red and orange signals mark the correlations of the glycerol backbone and head groups of co-purified lipids (40 ms mixing time), respectively. (PDF 2907 kb)

Additional file 5: **Figure S5.** ^13^C ssNMR spectra of liposomes. (a). ^13^C cross-polarization spectrum of pure DOPG liposomes, measured at 500 MHz (^1^H-frequency) using 10 kHz MAS. The black-dashed box corresponds to the glycerol-backbone and headgroup region between 60 and 80 ^13^C ppm, which is shown as a zoom in b). In b), the glycerol backbone and PG headgroup signals are indicated. These signals correspond well to the correlations observed in the 2D PARIS spectrum of MelB_St_. (b). ^13^C cross-polarization spectrum of mixed 9:1 DOPE:DOPG liposomes, measured at 400 MHz (^1^H-frequency) using 10 kHz MAS. The spectral region between 60 and 80 ^13^C ppm is shown. (PDF 740 kb)

Additional file 6: **Figure S6.** Identification of lipid species associated with purified MelB_St_ by HPLC-MS. Lipids extraction for HPLC-MS analyses and MelB_St_ delipidation treatment were carried out as described in Methods. (a). A typical base peak chromatogram of the separation of phospholipids co-purified with MelB_St_ protein. PG, CL, and PE peaks, as well as detergent UDM, are indicated. (b and c). PE and PG spectra before and after delipidation of MelB_St_. (PDF 990 kb)

Additional file 7: **Figure S7.** Lipid analyses by TLC. Lipids were extracted from purified MelB_St_ proteins before (100 μg) and after delipidation treatment (400 μg) as described in Methods. 70 μg of *E. coli* Extract Polar (Avanti Polar lipids INC) and 20 μg of individual lipids in CHCl_3_ were used as standards and directly spotted on the pre-treated TLC plates. Samples were run using an alkaline solvent system [CHCl_3_:MeOH: 28% NH_4_OH:H_2_O (45:35:1.6:8, *v*/v/*v*/*v*)]. (PDF 714 kb)

Additional file 8: **Figure S8.** Analyses of lipid chain length and degree of unsaturation. MelB_St_ protein samples before and after delipidation were subjected to HPLC-MS analyses, and lipid chain length and degree of unsaturation were analyzed. (a). The lipid acyl chain length is expressed as the total number of carbons per two fatty acyl chains. (b). Lipid unsaturation. Error bar, SEM; number of tests = 3. (PDF 239 kb)
