# Supplementary figures and images for: Structural and functional characterization of protein–lipid interactions of the Salmonella typhimurium melibiose transporter MelB
Source: BMC Biol. 2018 Aug 3;16:85. doi: 10.1186/s12915-018-0553-0 (PMC6091025; doi:10.1186/s12915-018-0553-0)

Additional File 1: Fig . S1

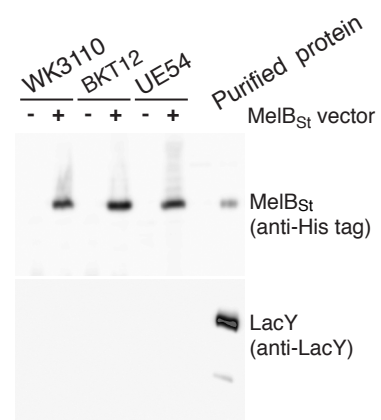

Supplement: Supplementary file 1 — Figure S1. Protein expression. Cells were grown in LB media containing 10 mM glucose at 30 °C for 5 h, and cell membranes were prepared. 20 μg of total membrane proteins were analyzed by SDS-15%PAGE and western blot. (a). MelBSt expression was detected by Penta⋅His HRP antibody. (b). An anti-C terminal LacY antibody was used to detect LacY expression. (PDF 864 kb) [file 12915_2018_553_MOESM1_ESM.pdf]

## Additional File 2: Fig . S2

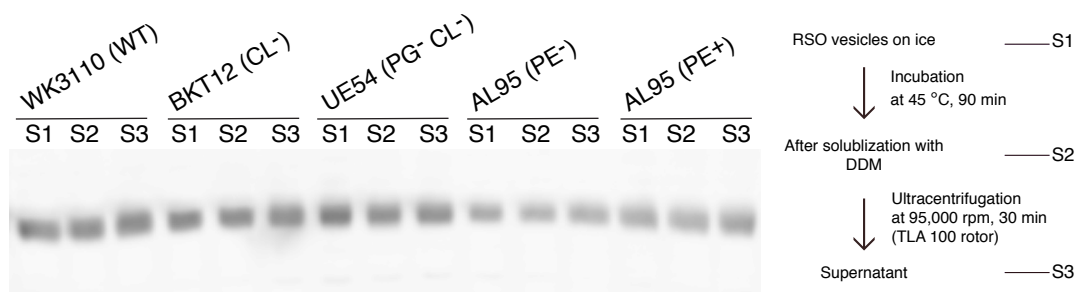

Supplement: Supplementary file 2 — Figure S2. MelBSt stability test in situ. RSO vesicles prepared from MelBSt-expressing cells with different lipid compositions (sample S1) were incubated at 45 °C for 90 min, and then solubilized with detergent DDM (sample S2). After separation by ultracentrifugation, the soluble MelBSt retaining in the supernatant (sample S3) was analyzed by SDS-15% PAGE and western blot using Penta⋅His HRP antibody. (PDF 135 kb) [file 12915_2018_553_MOESM2_ESM.pdf]

Additional File 3: Fig . S3

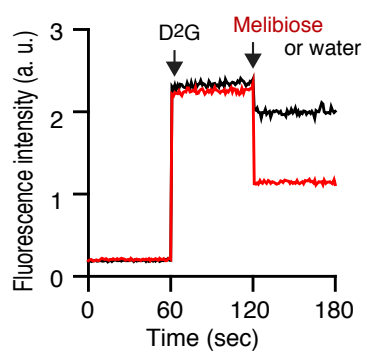

Supplement: Supplementary file 3 — Figure S3. Galactoside binding. A Trp→ D2G FRET assay was used to detect the binding of the [13C, 15N]-labeled MelBSt after reconstituted into proteoliposomes as described in the Methods. On the time trace set at an excitation wavelength of 290 nm and emission wavelength of 490 nm, D2G at 10 μM was added into the MelBSt liposome samples at 60-s time point, and melibiose at a saturation concentration or equal volume of water was further added into the solution at 120-s time point. (PDF 701 kb) [file 12915_2018_553_MOESM3_ESM.pdf]

Additional File 4: Fig . S4

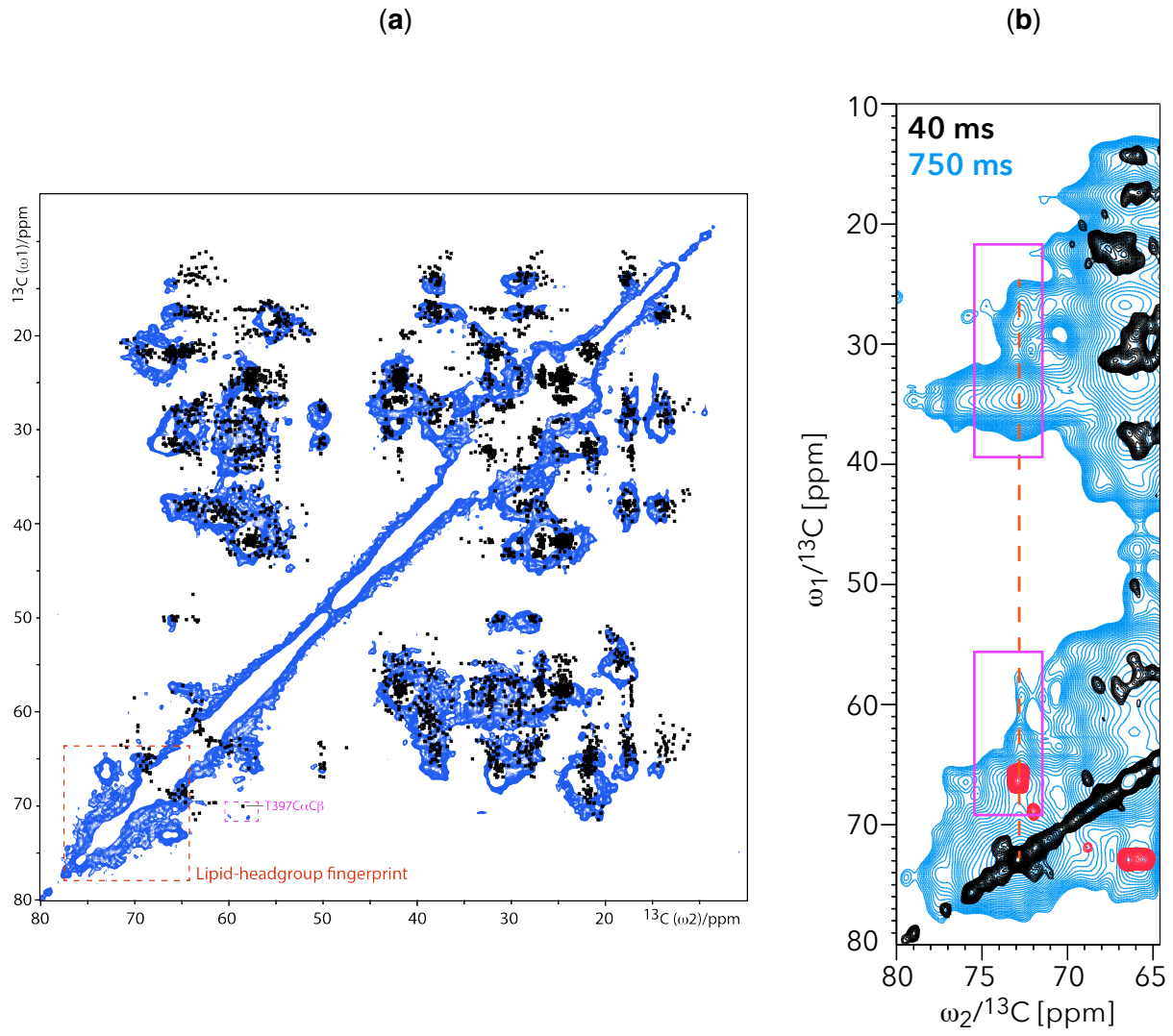

Supplement: Supplementary file 4 — Figure S4 (a) Overlay of the 2D 13C-13C PARIS spectrum with predictions derived from the 3D X-ray crystal structure of MelBSt. The 2D 13C-13C PARIS spectrum from Fig. 5 of the main text is superimposed with FANDAS [43] chemical shift predictions [69] derived from the MelBSt X-ray structure [PDB access ID, 4 M64]. Globally, the ssNMR signals match very well to the predictions. The headgroup signals of the co-purified lipids have no corresponding predictions from the protein. No lipid was resolved in the X-ray structure. (b) Specific contacts between MelBSt and lipid tail/glycerol-backbone. A 2D ssNMR PARIS-xy spectrum with a very long 13C-13C mixing time of 750 mx was measured at 250 K. The cross-peaks highlighted by magenta boxes are consistent with specific protein-lipid contacts. The red and orange signals mark the correlations of the glycerol backbone and head groups of co-purified lipids (40 ms mixing time), respectively. (PDF 2907 kb) [file 12915_2018_553_MOESM4_ESM.pdf]

Additional File 5: Fig . S5

(a) DOPG liposomes

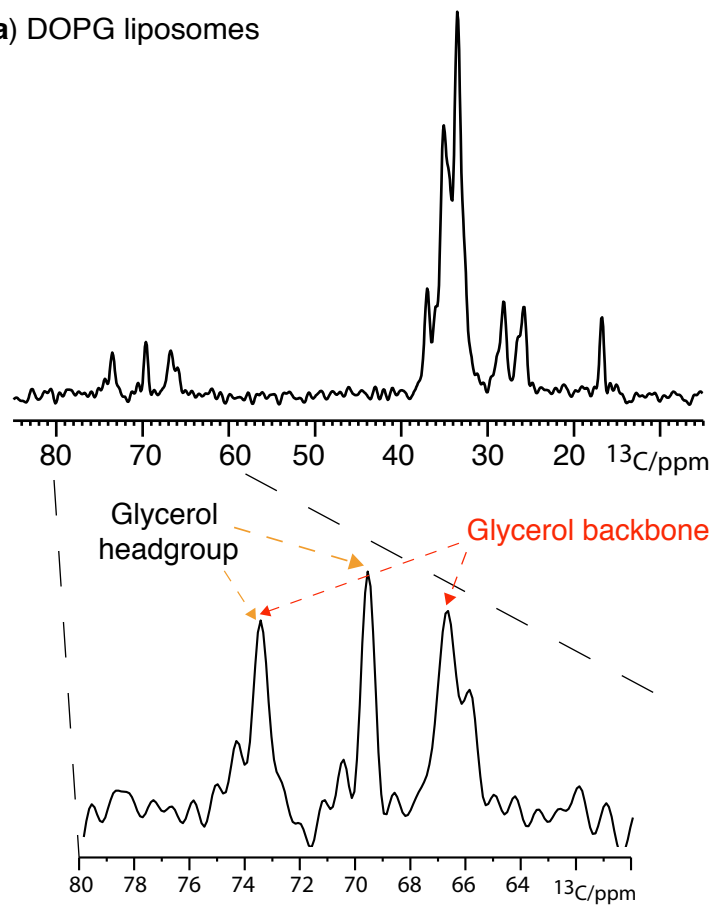

(b) DOPE:DOPG (9:1) liposomes

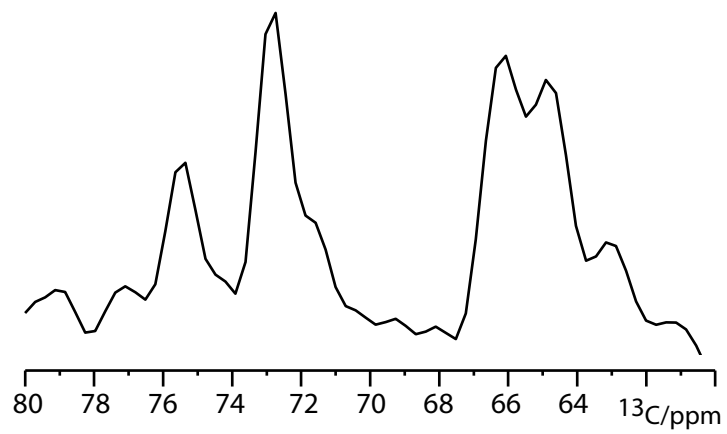

Supplement: Supplementary file 5 — Figure S5. 13C ssNMR spectra of liposomes. (a). 13C cross-polarization spectrum of pure DOPG liposomes, measured at 500 MHz (1H-frequency) using 10 kHz MAS. The black-dashed box corresponds to the glycerol-backbone and headgroup region between 60 and 80 13C ppm, which is shown as a zoom in b). In b), the glycerol backbone and PG headgroup signals are indicated. These signals correspond well to the correlations observed in the 2D PARIS spectrum of MelBSt. (b). 13C cross-polarization spectrum of mixed 9:1 DOPE:DOPG liposomes, measured at 400 MHz (1H-frequency) using 10 kHz MAS. The spectral region between 60 and 80 13C ppm is shown. (PDF 740 kb) [file 12915_2018_553_MOESM5_ESM.pdf]

Additional File 6: Fig . S6

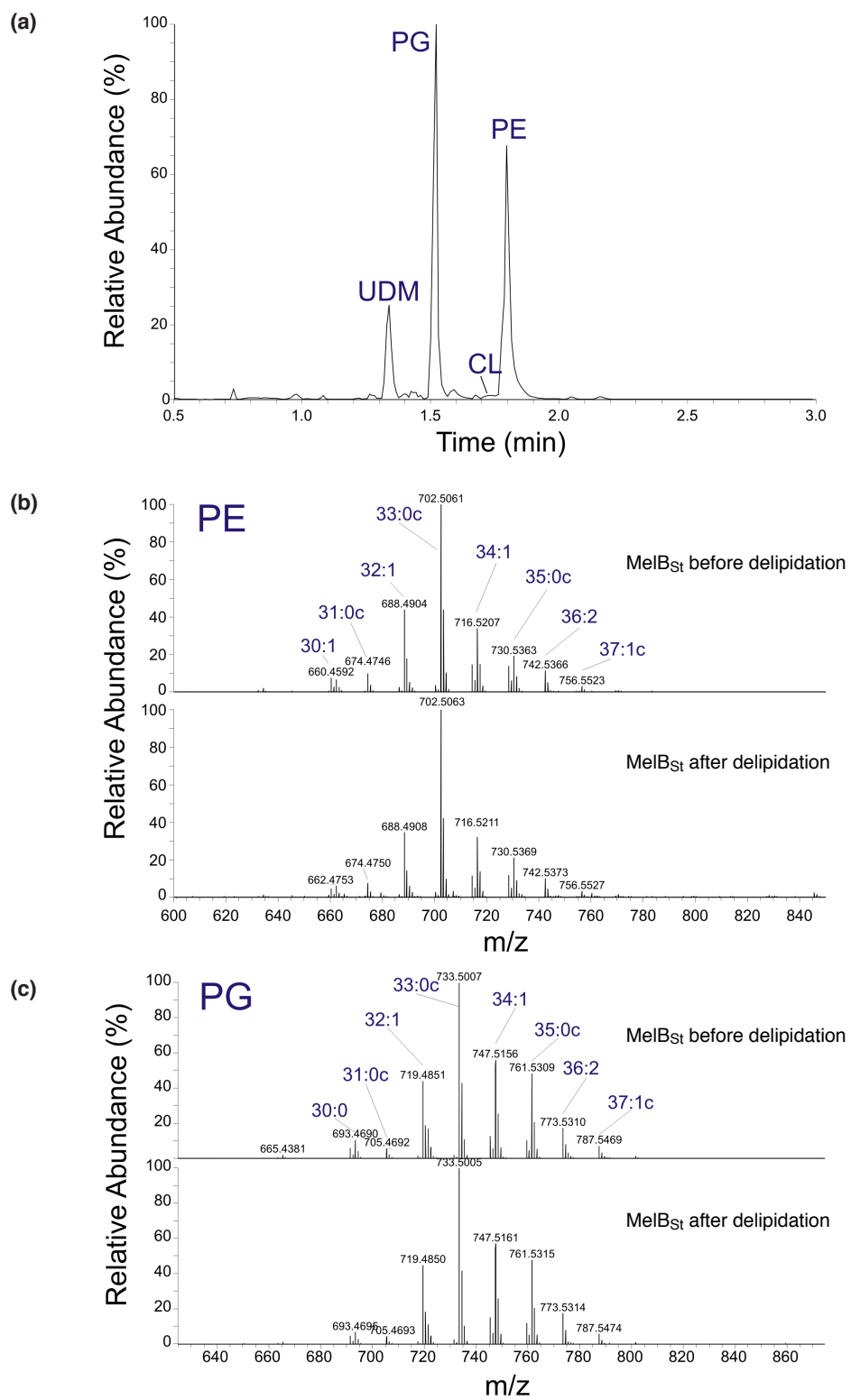

Supplement: Supplementary file 6 — Figure S6. Identification of lipid species associated with purified MelBSt by HPLC-MS. Lipids extraction for HPLC-MS analyses and MelBSt delipidation treatment were carried out as described in Methods. (a). A typical base peak chromatogram of the separation of phospholipids co-purified with MelBSt protein. PG, CL, and PE peaks, as well as detergent UDM, are indicated. (b and c). PE and PG spectra before and after delipidation of MelBSt. (PDF 990 kb) [file 12915_2018_553_MOESM6_ESM.pdf]

Additional File 7: Fig . S7

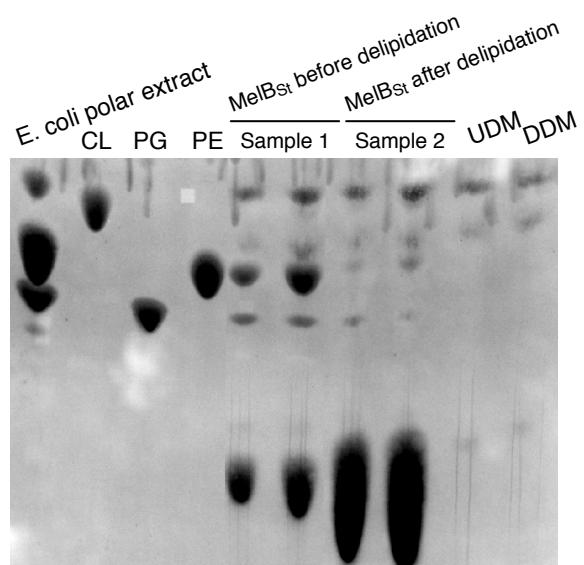

Supplement: Supplementary file 7 — Figure S7. Lipid analyses by TLC. Lipids were extracted from purified MelBSt proteins before (100 μg) and after delipidation treatment (400 μg) as described in Methods. 70 μg of E. coli Extract Polar (Avanti Polar lipids INC) and 20 μg of individual lipids in CHCl3 were used as standards and directly spotted on the pre-treated TLC plates. Samples were run using an alkaline solvent system [CHCl3:MeOH: 28% NH4OH:H2O (45:35:1.6:8, v/v/v/v)]. (PDF 714 kb) [file 12915_2018_553_MOESM7_ESM.pdf]

Additional File 8: Fig . S8

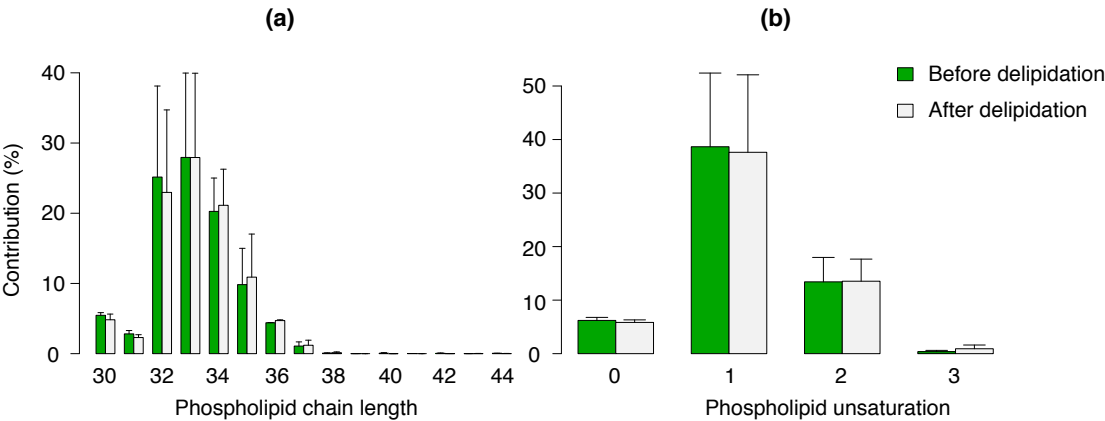

Supplement: Supplementary file 8 — Figure S8. Analyses of lipid chain length and degree of unsaturation. MelBSt protein samples before and after delipidation were subjected to HPLC-MS analyses, and lipid chain length and degree of unsaturation were analyzed. (a). The lipid acyl chain length is expressed as the total number of carbons per two fatty acyl chains. (b). Lipid unsaturation. Error bar, SEM; number of tests = 3. (PDF 239 kb) [file 12915_2018_553_MOESM8_ESM.pdf]
